# Vedolizumab Induces Remission in Two Cases of Ulcerative Colitis With Upper Gastrointestinal Involvement

**DOI:** 10.1002/deo2.70205

**Published:** 2025-09-03

**Authors:** Shinya Nakatani, Yuta Yamazaki, Kensuke Higuchi, Yumi Otoyama, Norihiro Suzuki, Kazuo Kikuchi, Takahisa Fujiwara, Atsushi Katagiri, Jyun Ohara, Hitoshi Yoshida

**Affiliations:** ^1^ Department of Medicine Division of Gastroenterology Showa Medical University School of Medicine Tokyo Japan; ^2^ Department of Pathology Showa Medical University School of Medicine Tokyo Japan

**Keywords:** case report, gastritis, ulcerative colitis, upper gastrointestinal tract, vedolizumab

## Abstract

Ulcerative colitis (UC) predominantly affects the colon; upper gastrointestinal involvement (UGI) has been reported, but no established treatments exist. We report two cases of UC with concomitant UGI that showed positive responses to vedolizumab therapy. Case 1 involved a 29‐year‐old man who developed continuous inflammation extending from the stomach to the jejunum 1 month after an initial UC diagnosis. Intravenous prednisolone provided clinical remission; however, maintenance therapy with oral azathioprine was unsuccessful. Vedolizumab was initiated. Three months later, esophagogastroduodenoscopy (EGD) and colonoscopy confirmed the resolution of inflammation. Case 2 involved a 19‐year‐old man diagnosed with UGI via endoscopy while being evaluated for nausea and fever during UC treatment. 5‐aminosalicylic acid and prednisolone therapies were ineffective; therefore, vedolizumab was administered. Three months later, EGD confirmed mucosal healing. Both patients have maintained clinical remission for >2 years. To our knowledge, this is the first report of UC with UGI involvement that was successfully treated with vedolizumab. These findings suggest that vedolizumab is effective and safe for UGI treatment.

## 1 Introduction

Ulcerative colitis (UC) is a chronic inflammatory disease that predominantly affects the mucosal and submucosal layers of the colon and rectum [1]. Although its pathogenesis remains unclear, it is believed to result from dysregulated immune responses triggered by environmental and genetic factors [1].

Rare cases of UC‐related upper gastrointestinal involvement (UGI) have been reported (8% and 3%–10% in the stomach and duodenum, respectively) [2]. Hisabe et al. [3] reported UGI inflammation in 6.2% of patients with pancolitis and 4% of patients who had undergone colectomy. UGI may be underdiagnosed owing to nonspecific symptoms, including nausea and epigastric pain [4].

No standard treatment for UGI exists; however, 5‐aminosalicylic acid, corticosteroids, anti‐tumor necrosis factor‐alpha (anti‐TNF‐α) agents, and calcineurin inhibitors have been efficacious [4]. Recent reports have suggested that upadacitinib may also be effective in treating postoperative inflammatory enteropathies [5]. However, vedolizumab is considered efficacious for treating UGI. We present two cases of UC with UGI that were successfully treated with vedolizumab.

## 2 Case Report

### 2.1 Case 1

A 29‐year‐old man who had undergone lower gastrointestinal endoscopy due to bloody and watery diarrhea (*>*6 times a day) was diagnosed with pancolitis‐type UC and received 5‐ASA therapy.

One month later, he presented to us with abdominal pain. His C‐reactive protein (CRP) level was 2.70 mg/dL. Considering the possibility of 5‐ASA intolerance, this therapy was discontinued. However, persistent abdominal pain and nausea prompted endoscopy.

**FIGURE 1:**
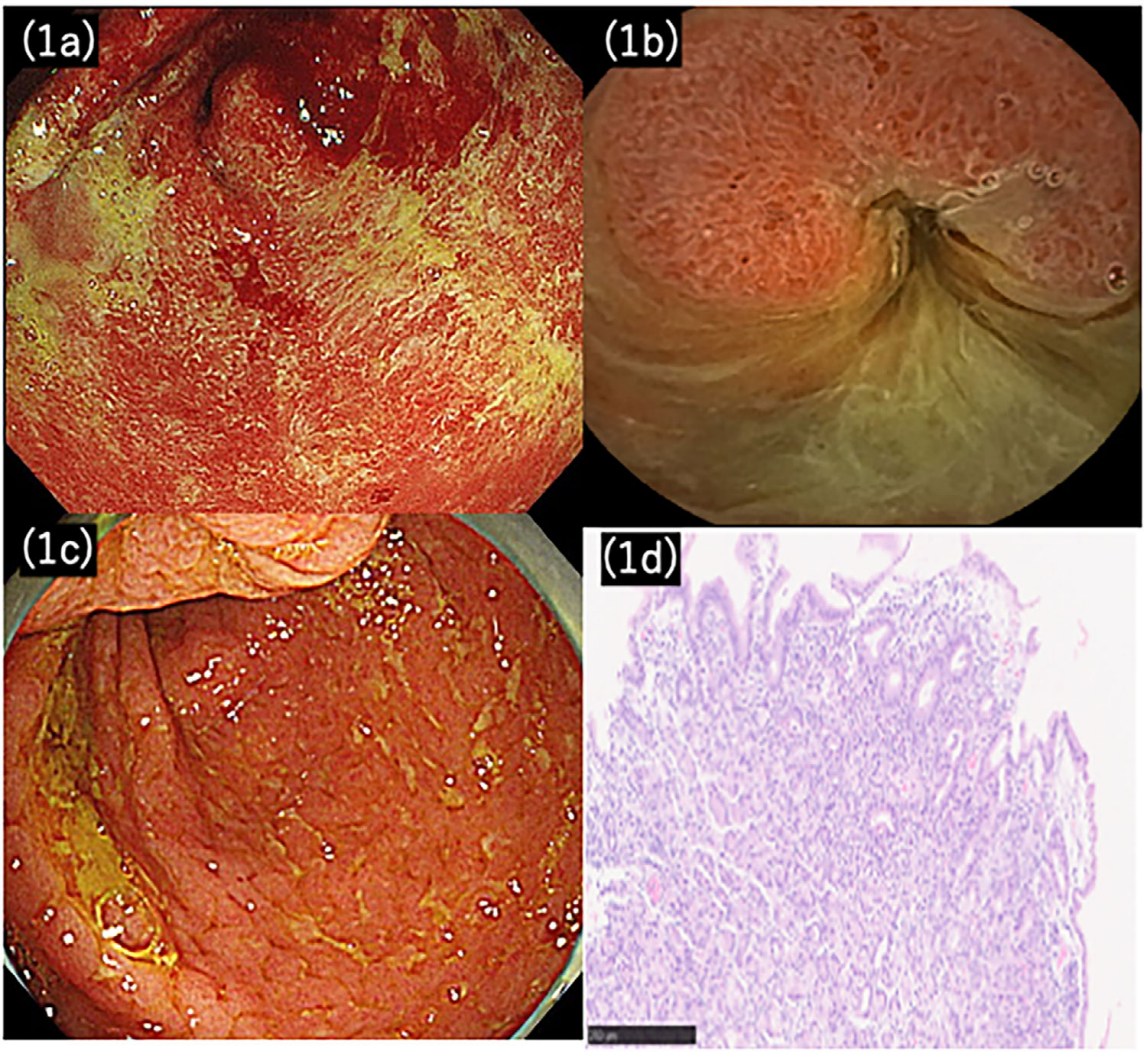
Initial findings in Case 1. Upper gastrointestinal endoscopy showing edematous wall thickening and coarse friable mucosa with contact bleeding in the stomach (a). Capsule endoscopy showing continuous inflammation from the duodenum to the proximal jejunum with prominent edematous changes in the proximal jejunum (b). Colonoscopy revealed mild inflammation throughout the colon (Mayo endoscopic subscore of 2) (c). Histopathological findings showing marked neutrophilic infiltration in the lamina propria with involvement of glandular epithelium (cryptitis) and formation of crypt abscesses. Lymphocytes and scattered eosinophils are also present, consistent with UC‐related upper gastrointestinal involvement presenting as focally enhanced gastritis (H&E stain) (d).

Esophagogastroduodenoscopy (EGD) revealed continuous diffuse coarse mucosa with purulent exudates, and capsule endoscopy revealed erythema extending into the jejunum (Figure 1a,b).

The *Helicobacter pylori* test was negative. Colonoscopy revealed moderate inflammation throughout the colon with Mayo endoscopic subscore (MES) 2 (Figure 1c).

Histopathology of a gastric biopsy revealed marked neutrophilic infiltration in the lamina propria, together with lymphocytic and eosinophilic infiltration, cryptitis, and crypt abscesses, consistent with UC (Figure 1d). Thus, the patient was diagnosed with UGI. On day 6, an oral H2 blocker and intravenous prednisolone (40 mg) were administered, which led to marked clinical improvement. The patient was transitioned to outpatient care on day 20, and prednisolone was discontinued after 2 months. Azathioprine (50 mg) was introduced for maintenance, and H2 blocker therapy was continued. The patient's condition remained stable, and EGD and colonoscopy confirmed remission 3 months after prednisolone initiation.

**FIGURE 2:**
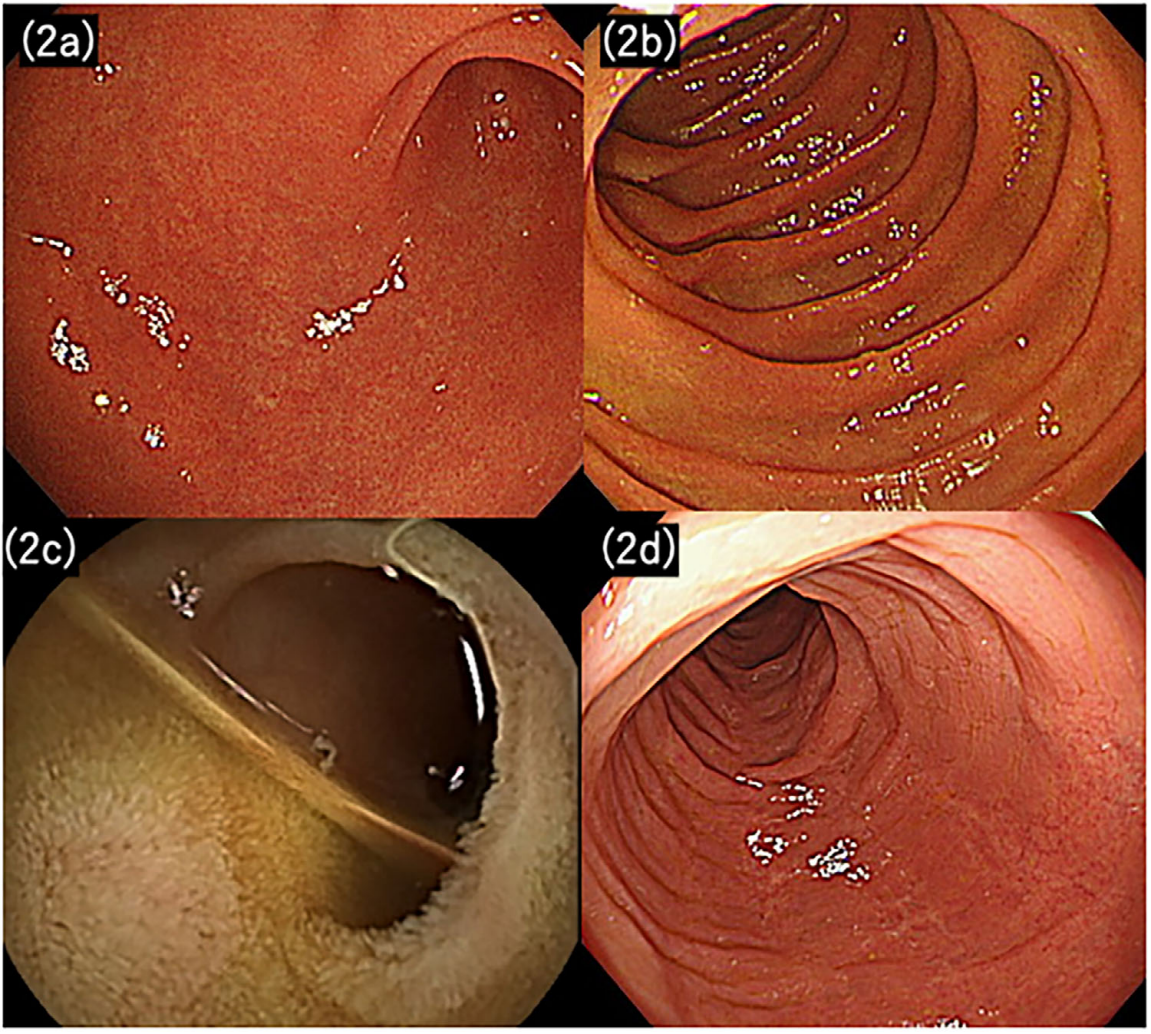
Findings in Case 1 at 3 months after vedolizumab initiation. Inflammation had improved in the stomach (a), duodenum (b), jejunum (c), and colon (d). No evidence of atrophic gastritis was observed.

Nine months after prednisolone initiation, the patient relapsed with epigastric pain and watery diarrhea (*>*6 times a day). EGD revealed recurrent gastric wall thickening and erosion, whereas colonoscopy showed pancolitis (MES 2). H2 blockers were considered ineffective. Based on the steroid dependence of UC and UGI, maintenance of remission with biologic or Janus kinase (JAK) inhibitor therapy was deemed necessary. After shared decision‐making, the patient selected vedolizumab, given its favorable safety profile. Two weeks after starting vedolizumab, abdominal pain, nausea, and diarrhea resolved. Three months later, endoscopic and histological remission was confirmed on EGD (Figure 2a–c) and colonoscopy (Figure 2d). With 2‐monthly vedolizumab therapy, he has remained in remission for >4 years.

### 2.2 Case 2

A 19‐year‐old man with a history of depression presented to our hospital with watery diarrhea (*>*6 times a day) and persistent fever exceeding 37.5°C for 3 months. Based on previous endoscopic findings, he was diagnosed with pancolitis‐type UC and referred to our hospital. Laboratory findings showed CRP corresponding to 2.06 mg/dL. Severe nausea and poor food intake prompted hospitalization.

**FIGURE 3:**
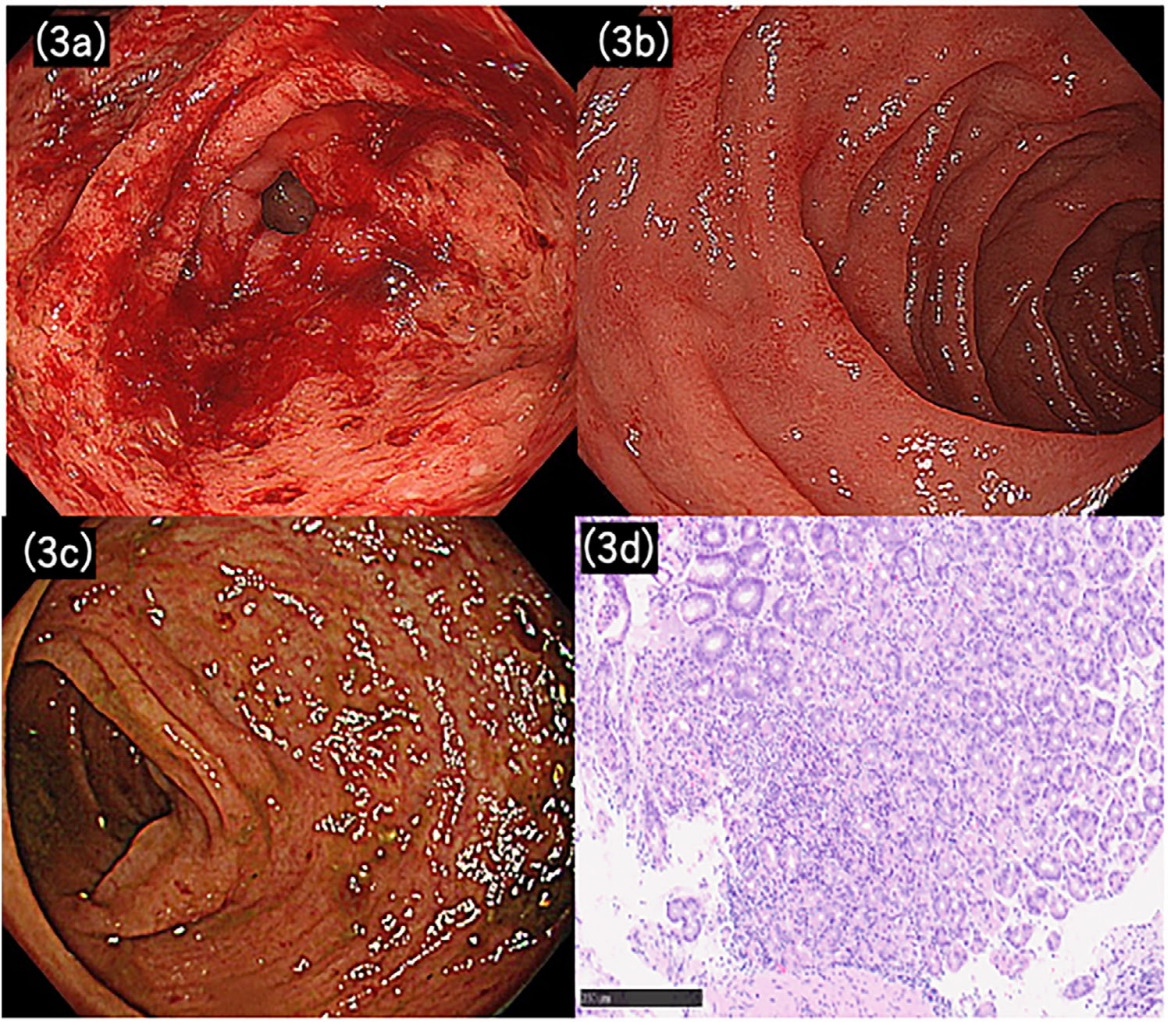
Initial findings in Case 2. Upper gastrointestinal endoscopy revealed continuous edematous wall thickening from the stomach (a) to the duodenum (b). The gastric mucosa was friable and prone to bleeding. Colonoscopy revealed mild inflammation (Mayo endoscopic subscore of 1) (c). Gastric biopsy specimen from Case 2 showing focally enhanced gastritis, characterized by predominant neutrophilic infiltration with admixed lymphocytes and eosinophils within the lamina propria and crypt epithelium (H&E stain) (d).

On day 1 of hospitalization, he received oral mesalazine (4000 mg), an intravenous proton pump inhibitor (PPI), and intravenous prednisolone (40 mg). The inflammation (CRP: 1.84 mg/dL) became minimal, but the nausea persisted. On day 6, EGD revealed continuous mucosal edema and erythema from the stomach to the duodenum(Figure 3a,b). The gastric mucosa was friable, with mild bleeding. Histology revealed fundic glands with minimal atypia and focal infiltration of neutrophils, eosinophils, and lymphocytes in the lamina propria, consistent with focally enhanced gastritis (Figure 3d). Colonoscopy revealed mild inflammation throughout the colon (MES 1) (Figure 3c). On day 8, blood tests showed CRP levels of 1.46 mg/dL. Nausea persisted without improvement. *Helicobacter pylori* test results were negative.

**FIGURE 4:**
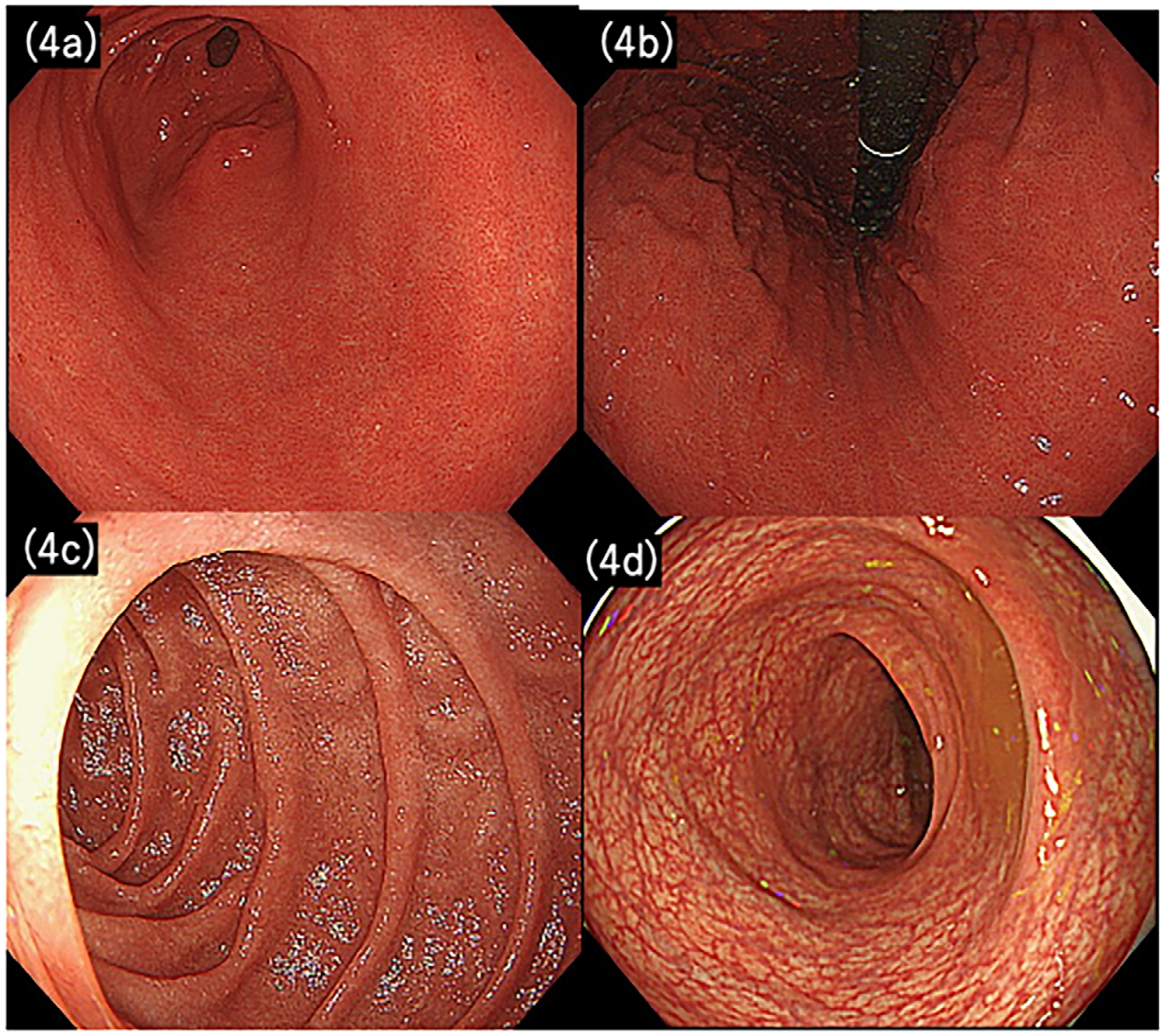
Findings in Case 2 at 3 months after vedolizumab initiation. Inflammation had improved in the stomach (a,b) and duodenum (c), and no colonic inflammation was detected (d).

The patient was diagnosed with steroid‐ and PPI‐refractory UC with UGI. After shared decision‐making regarding biologic/JAK, the patient selected vedolizumab, given its favorable safety profile. By day 3 of vedolizumab therapy, the fever resolved. The nausea subsided, and oral intake was resumed. The patient was discharged on day 6 of vedolizumab therapy and followed up as an outpatient. Laboratory findings showed improvement 3 months later (i.e., CRP: 0.07 mg/dL). EGD showed complete resolution of inflammation in the stomach and duodenum, with histological confirmation of remission (Figure 4a–c). Colonoscopy was initially not performed due to a lack of consent. However, the patient remained asymptomatic and was judged to be in clinical remission. Consent for colonoscopy was obtained 2 years after vedolizumab initiation, and the examination confirmed endoscopic remission (Figure 4d).

## 3 Discussion

To our knowledge, this is the first report of non‐operative cases of UGI complicated with UC from the initial presentation, and the first cases in which vedolizumab induced long‐term remission of extensive gastrointestinal lesions, involving the stomach, duodenum, and jejunum.

UGI is typically diagnosed by a combination of clinical context, upper endoscopic continuity of inflammation, and characteristic histopathological findings such as cryptitis, crypt abscesses, and neutrophilic infiltration of the lamina propria [1, 2, 3]. These features must be distinguished from drug‐induced, infectious, and eosinophilic gastrointestinal diseases to ensure accurate diagnosis [2, 4]. Both patients were *H. pylori*‐negative, had no relevant medication history, and improved only after vedolizumab, not after mesalamine withdrawal. Endoscopy showed continuous mucosal inflammation from the stomach to the duodenum, consistent with UGI rather than focal drug‐induced or infectious lesions. Cryptitis, crypt abscesses, and neutrophilic infiltration were observed histologically. In Case 2, histology showed focally enhanced gastritis, a localized inflammatory pattern frequently reported in UC‐related UGI [4]. Although eosinophils were present focally, the overall features supported UC.

Notably, both cases demonstrated resolution of inflammation in the stomach and duodenum following vedolizumab therapy. Vedolizumab exerts its gut‐selective anti‐inflammatory effect by blocking the interaction between α4β7 integrin on lymphocytes and mucosal addressin cell adhesion molecule‐1 (MAdCAM‐1) on vascular endothelial cells. Recent studies [6] have shown that eosinophils, such as lymphocytes, also express α4β7 integrin and can migrate into the gastrointestinal mucosa through MAdCAM‐1–mediated pathways. In our cases, although histology predominantly revealed neutrophilic infiltration, focal eosinophilic infiltration was also observed, contributing to the observed vedolizumab responsiveness (Figure S1a,b).

Additional immunohistochemical staining performed for this study revealed MAdCAM‐1 expression on vascular endothelial cells in the stomach and duodenum (Figure S1c,d). Although MAdCAM‐1 expression in the gastric mucosa is limited, reports have suggested that such ectopic expression can be induced by chronic inflammation [7, 8]. Further, vedolizumab has shown efficacy in Ménétrier's disease—a condition with prominent gastric involvement—and chronic pouchitis, where clinical remission rates reach 50%–60% [9, 10]. Although pouchitis and UGI are distinct entities, these data suggest that vedolizumab may be effective in controlling UC‐like inflammation beyond the colon.

In both cases, anti‐TNF‐α therapy—previously reported as effective for UGI—was declined by the patients due to safety concerns. Given the moderate disease activity, we selected vedolizumab through shared decision‐making, opting to escalate to anti‐TNF‐α therapy if necessary. The choice was further supported by vedolizumab's favorable safety profile and its gut‐selective mechanism of action via α4β7 integrin blockade, which we hypothesized could be effective in UGI based on previous reports and the observed MAdCAM‐1 expression in our cases.

These cases have limitations. First, vedolizumab was selected because anti‐TNF‐α therapy was declined due to safety concerns. However, compared with other agents that have been reported to be effective in previous studies, it remains unclear whether vedolizumab provides superior efficacy. Second, the significance of MAdCAM‐1 expression in the gastric mucosa remains unclear, given the limited prior data; whether it reflects ectopic inflammation or predicts vedolizumab response warrants further study. Third, concurrent UC and UGI onset is rare, and the clinical behavior and treatment response in non‐operative cases remain unclear, particularly compared with postoperative UGI.

In conclusion, these cases underscore the importance of early recognition and prompt endoscopic evaluation of UGI in patients with UC. When conventional therapy is insufficient, biologic agents—including vedolizumab—may be considered, especially in bio‐naïve cases, given its favorable safety profile.

## Conflicts of Interest

The authors declare no conflicts of interest.

## Ethics Statement

All the procedures were performed in accordance with the ethical standards of the 1964 Declaration of Helsinki and its later amendments. Written informed consent was obtained from both patients for publication of this case report and accompanying images. This manuscript was prepared in accordance with CARE (CAseREport) guidelines.

## Clinical Trial Registration

N/A

## Supporting information




**FIGURE S1** Histological findings in both cases. (a) Case 1: Eosinophilic infiltration in the vascular endothelium of the gastric mucosa (H&E stain). (b) Case 2: Eosinophilic infiltration in the vascular endothelium of the duodenal mucosa (H&E stain). (c) Case 1: Positive MAdCAM‐1 immunostaining in vascular endothelial cells of the gastric mucosa. (d) Case 2: Positive MAdCAM‐1 immunostaining in vascular endothelial cells of the duodenal mucosa.
