# Supplementary figures and images for: Vedolizumab Induces Remission in Two Cases of Ulcerative Colitis With Upper Gastrointestinal Involvement
Source: DEN Open. 2025 Sep 3;6(1):e70205. doi: 10.1002/deo2.70205 (PMC12408375; doi:10.1002/deo2.70205)

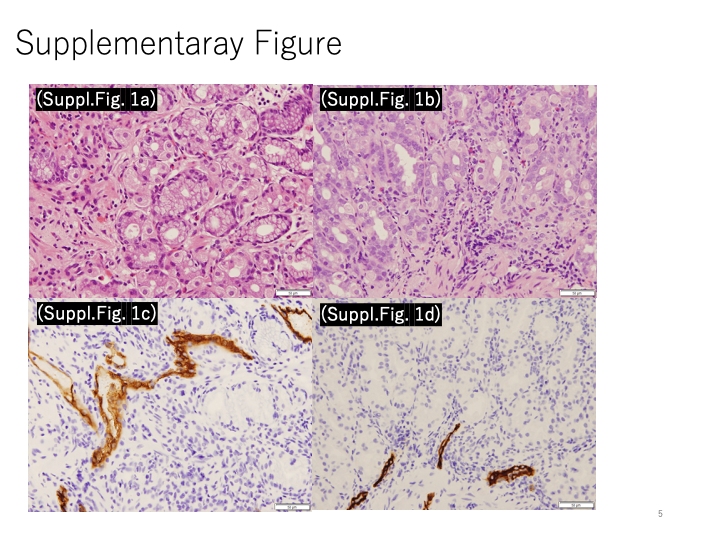

Supplement: Supplementary file 1 — FIGURE S1 Histological findings in both cases. (a) Case 1: Eosinophilic infiltration in the vascular endothelium of the gastric mucosa (H&E stain). (b) Case 2: Eosinophilic infiltration in the vascular endothelium of the duodenal mucosa (H&E stain). (c) Case 1: Positive MAdCAM‐1 immunostaining in vascular endothelial cells of the gastric mucosa. (d) Case 2: Positive MAdCAM‐1 immunostaining in vascular endothelial cells of the duodenal mucosa. [file DEO2-6-e70205-s001.tiff]
